# Initial experience with radiomics of carotid perivascular adipose tissue in identifying symptomatic plaque

**DOI:** 10.3389/fneur.2024.1340202

**Published:** 2024-02-16

**Authors:** Ji-Yan Nie, Wen-Xi Chen, Zhi Zhu, Ming-Yu Zhang, Yu-Jin Zheng, Qing-De Wu

**Affiliations:** ^1^Department of Radiology, ShunDe Hospital of Guangzhou University of Chinese Medicine, Foshan, China; ^2^Graduate School, Guangzhou University of Chinese Medicine, Guangzhou, China

**Keywords:** carotid atherosclerosis, perivascular adipose tissue, radiomics, stroke, transient ischemic attack

## Abstract

**Background:**

Carotid atherosclerotic ischemic stroke threatens human health and life. The aim of this study is to establish a radiomics model of perivascular adipose tissue (PVAT) around carotid plaque for evaluation of the association between Peri-carotid Adipose Tissue structural changes with stroke and transient ischemic attack.

**Methods:**

A total of 203 patients underwent head and neck computed tomography angiography examination in our hospital. All patients were divided into a symptomatic group (71 cases) and an asymptomatic group (132 cases) according to whether they had acute/subacute stroke or transient ischemic attack. The radiomic signature (RS) of carotid plaque PVAT was extracted, and the minimum redundancy maximum correlation, recursive feature elimination, and linear discriminant analysis algorithms were used for feature screening and dimensionality reduction.

**Results:**

It was found that the RS model achieved the best diagnostic performance in the Bagging Decision Tree algorithm, and the training set (AUC, 0.837; 95%CI: 0.775, 0.899), testing set (AUC, 0.834; 95%CI: 0.685, 0.982). Compared with the traditional feature model, the RS model significantly improved the diagnostic efficacy for identifying symptomatic plaques in the testing set (AUC: 0.834 vs. 0.593; Z = 2.114, *p* = 0.0345).

**Conclusion:**

The RS model of PVAT of carotid plaque can be used as an objective indicator to evaluate the risk of plaque and provide a basis for risk stratification of carotid atherosclerotic disease.

## 1 Introduction

Carotid atherosclerotic disease is the main cause of ischemic stroke, accounting for about 34% of ischemic stroke (1). The guidelines for the prevention and treatment of Stroke in China 2021 recommend carotid endarterectomy or carotid artery stenting for patients with more than 50% carotid artery stenosis to prevent stroke. However, the degree of carotid artery stenosis does not completely match the occurrence of stroke (2), and there is currently a lack of objective indicators to assess the risk of stroke in carotid plaque. Head and neck computed tomography angiography (CTA) is the first line non-invasive imaging method for carotid atherosclerosis (3). Radiomics analysis of carotid plaques based on CTA has made some progress in identifying carotid plaques at high risk of stroke. However, automatic segmentation of carotid plaques is challenging due to the complex composition of plaques and the limited number of pixels in CTA images. As a consequence, the radiomic signature (RS) model derived from these segmentations often exhibits low performance and lacks universality (4). Vascular inflammation can drive atherosclerotic plaque rupture and thrombosis, leading to the occurrence of adverse cardiovascular and cerebrovascular events (5). A considerable body of recent research (6–10) has demonstrated that perivascular adipose tissue (PVAT) can be automatically segmented by applying a threshold range of −190 to -30HU on CTA, enabling the monitoring of vascular inflammation and identification of symptomatic plaques. Numerous studies (11–14) have also indicated that the pericoronary adipose tissue RS model exhibits excellent performance in identifying and predicting symptomatic plaques; however, there is limited literature available regarding carotid artery investigations.

In this study, we used radiomics analysis combined with machine learning methods to establish an RS model based on the PVAT of carotid plaques combined with traditional patient characteristics and investigated its performance in distinguishing symptomatic and asymptomatic carotid plaques.

## 2 Materials and methods

### 2.1 Study population

**Figure 1:**
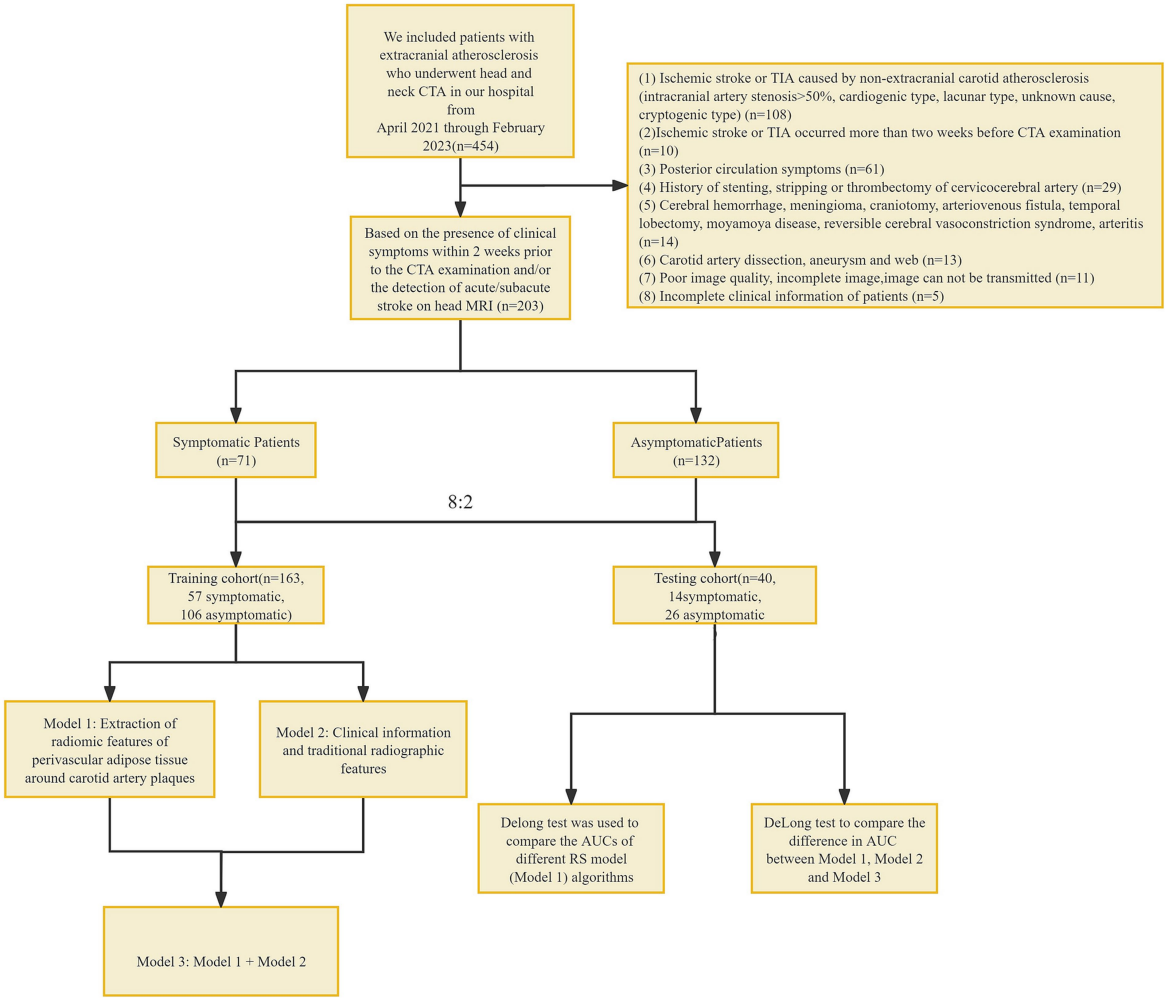
Flowchart. CTA, computed tomography angiography; TIA, transient ischemic attack, RS, radiomic signature.

This was a retrospective study involving patients who underwent head and neck CTA at our hospital from April 2021 through February 2023 (Figure 1). All patients were divided into a symptomatic group and an asymptomatic group according to whether they had clinical symptoms within 2 weeks before CTA examination and/or whether a head MRI showed acute/subacute stroke (15). Clinical symptoms included classic TIA (transient ischemic attack) and anterior circulation (carotid territory) ischemic stroke, as well as monocular symptoms ipsilateral (16) to the carotid plaque (amaurosis or retinal artery occlusion). Classic TIA is defined as an abnormal focal neurological deficit lasting less than 24 h. Complete ischemic stroke presents with the sudden onset of a focal neurologic deficit lasting >24 h (17). The patient’s age, gender, body mass index (BMI), history of hypertension, diabetes, hyperlipidemia, smoking history, history of antihypertensive drugs, and history of antiplatelet drugs were collected.

Inclusion criteria: (1) extracranial carotid atherosclerosis; Exclusion criteria: (1) ischemic stroke or TIA caused by non-extracranial carotid atherosclerosis (intracranial arterial stenosis >50%, cardiogenic type, lacunar type, unknown cause, cryptogenic type) (18–24); (2) ischemic stroke or TIA occurred more than 2 weeks before CTA examination; (3) posterior circulation symptoms; (4) history of stenting, stripping or thrombectomy of cervicocerebral artery; (5) Cerebral hemorrhage, meningioma, craniotomy, arteriovenous fistula, temporal lobectomy, moyamoya disease, reversible cerebral vasoconstriction syndrome, arteritis; (6) Carotid artery dissection, aneurysm and web; (7) poor image quality, incomplete image, image cannot be transmitted; and (8) incomplete clinical information of patients. This study has been approved by the Ethics Committee of Shunde Hospital, Guangzhou University of Traditional Chinese Medicine (Ethics Review approval: KY-2022010).

### 2.2 CT scanning parameters

Head and neck CTA was performed using a third-generation dual-source CT (Somatom Force, Siemens). The patient was placed in a supine position with head advanced and calm breathing. The scanning direction was the foot–head direction, and the scanning range was from the level of the sternal Angle to the skull dome. A measure of 50 mL of ioversol (Bayer, Germany, iodine concentration 370 mg/mL) was injected via the cubital vein with a high pressure syringe at a rate of 5 mL/s, and 40 mL of normal saline was injected at the same flow rate. The ROI was drawn at the descending aortic arch using contrast agent tracking technology. The trigger threshold was 100HU, and the scan was delayed for 3–4 s after the trigger. The tube voltage was 90–100 KVp, and the tube current was adaptive.

### 2.3 Plaque data analysis

All CTA data were transferred to head and neck CTA AI system (Shukun Technology, Beijing, China) for plaque localization and analysis on curved planar reconstruction images. The symptomatic group selected the narrowest carotid plaque on the symptomatic side, and the asymptomatic group selected the narrowest carotid plaque. According to the location of the plaque, the plaque was divided into left carotid artery plaque and right carotid artery plaque. The degree of plaque stenosis was automatically calculated.

Plaque thickness was measured as the maximum axial size of the plaque on a single axial slice, representing its maximum thickness. Plaque length was defined as the distance from the origin of the plaque to the distal end. The remodeling index was calculated by averaging the maximum external vessel diameter of the plaque over the normal diameter of the proximal and distal regions.

Plaques were classified into three types based on the presence or absence of calcification: calcified plaque, non-calcified plaque, and mixed plaque. The presence of plaque ulceration was identified by the spread of a contrast agent deep into the plaque on multiple slices from different imaging perspectives. High-risk plaque is defined as having two or more of the following features: positive remodeling index >1.1, punctate calcification (with a diameter < 3 mm, occupying <1/4 of the lumen’s diameter, and a CT value >130HU), low-density plaque (a non-calcified plaque with a CT value <30HU and an area of 1mm^2^ within the plaque), and the napkin ring sign (a contrast agent ring encircling a low-density plaque component, along with contrast agent in the surrounding vascular lumen).

### 2.4 Segmentation of plaque PVAT

**Figure 2:**
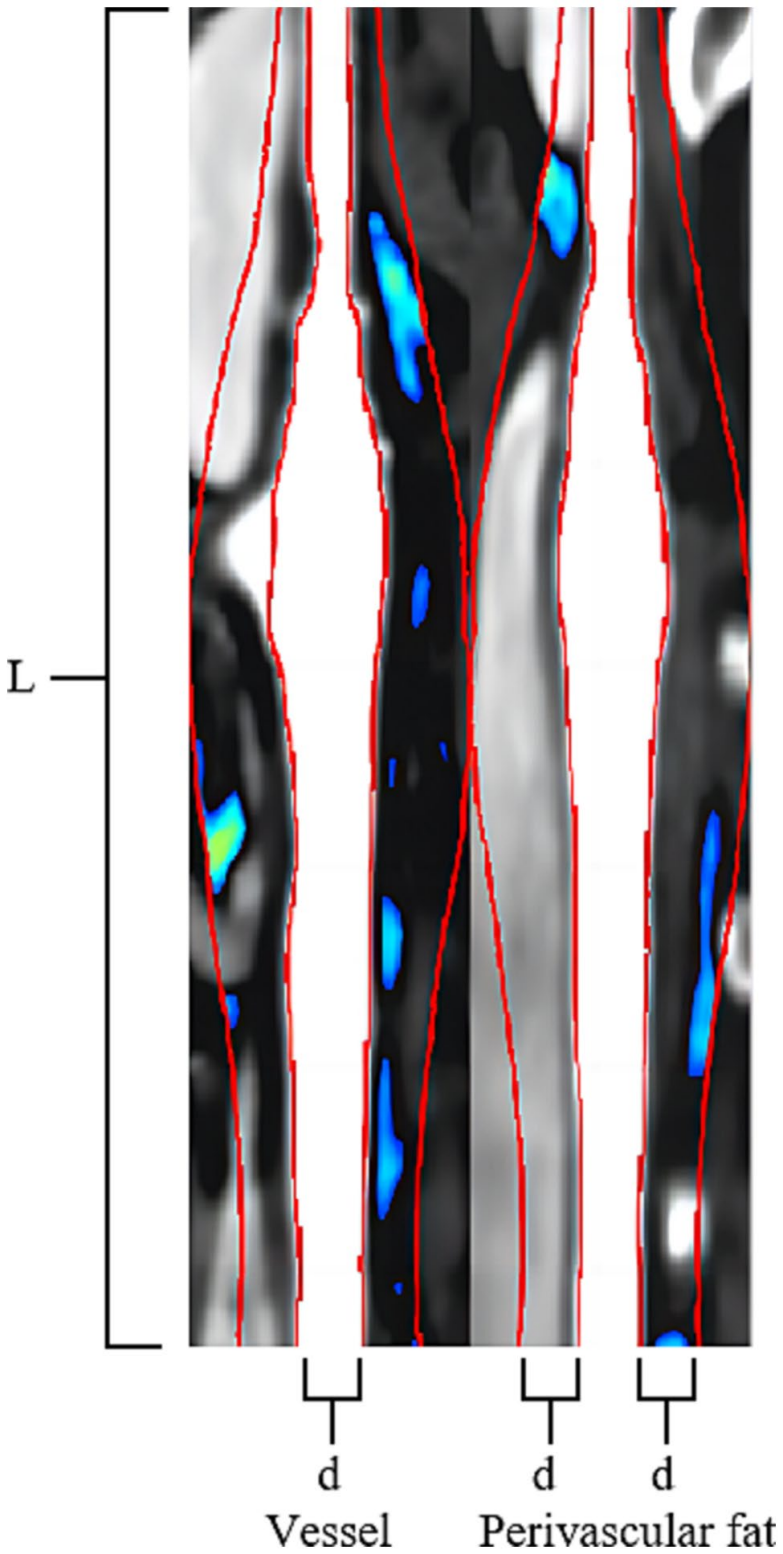
Carotid Plaque PVAT Segmentation Image. The Carotid Artery Straightening Image reveals a calcified plaque located at the bifurcation of the carotid artery. The red line was centered on the carotid bifurcation, extending 2 cm in the superior and inferior directions for a total length of 4 cm (L). The PVAT width of two red lines is equivalent to the diameter of the carotid artery beyond the outer wall of the carotid artery vessel (d). The software automatically segmented adipose tissue with an attenuation value of −190 HU to −30 HU along the target length and the width of the carotid artery vessel is visualized using a blue-green pseudocolored map.

ROI segmentation of the PVAT of extracranial carotid plaques was performed using perivascular fat analysis software (Shukun Technology, Beijing, China). The measurement was centered on the carotid bifurcation, extending 2 cm in the superior and inferior directions for a total length of 4 cm. The PVAT width was equivalent to the diameter of the carotid artery beyond the outer wall of the carotid artery vessel. The software automatically segmented adipose tissue with an attenuation value of −190 HU to −30 HU along the target length and width of the carotid artery vessel (25, 26) (Figure 2).

#### 2.4.1 Fat attenuation index analysis of plaques

The Fat Attenuation Index (FAI) surrounding atherosclerotic plaques was assessed using specialized perivascular fat analysis software (Shukun Technology, Beijing, China). The length of “Stenosis FAI” is measured on the narrowest cross-sectional slice of the plaque, while the length of “Stenosis range FAI” is measured along the entire extent of the plaque, from its origin to the distal end. Both FAI measurements have a width equivalent to the diameter of the carotid artery beyond the outer wall of the carotid artery vessel. The software automatically segmented adipose tissue with an attenuation value of −190 HU to −30 HU along the target length and width of the carotid artery vessel, following which the software automatically computes the average density of the perivascular fat encompassing the plaque (Figure 2).

### 2.5 RS extraction and selection of plaque PVAT

#### 2.5.1 RS extraction

ROI of all plaque PVAT was imported into Shukun AI Scientific Research Platform (Beijing, China) for RS extraction. A total of 1874 RS were extracted from the ROI of each plaque PVAT. These included 360 first-order features, 14 shape features, 480 gray level co-occurrence matrix (GLCM), 280 gray level dependence matrix (GLDM), 320 gray level run length matrix (GLRLM), 320 gray level size zone matrix (GLSZM), and 100 neighborhood gray tone difference matrix (NGTDM).

#### 2.5.2 RS selection and model construction

All the extracted features were imported into uAI Research Portal software (version 1.1, United, China) for feature selection and model construction. (1) The minimum redundancy maximum relevance (MRMR) algorithm is utilized to calculate the redundancy and relevance between each feature and the target variable, symptomatic plaques. Subsequently, 100 features are selected. A recursive feature elimination (RFE) algorithm selects feature subset by eliminating features with small contributions to prediction ability step by step. It determines the most important features for the prediction task by recursively training the model and evaluating feature importance. The RFE has been applied to select the most important features, resulting in 48 features, comprising 16 first-order features, 7 CLCM features, 6 GLDM features, 3 GLRLM features, 10 GLSZM features, 4 NGTDM features, and 2 shape features. (2) Linear discriminant analysis (LDA) is employed to reduce the dimensionality of the selected imaging features to 20 target dimensions. By using an mRMR algorithm, RFE algorithm, and LDA together, we can achieve several objectives: improving the effectiveness of feature selection, removing redundant and noisy features, extracting features with high classification ability, and reducing dimensionality. Through the comprehensive application of these methods, more robust and superior feature subsets can be obtained, which is helpful for better image-based radiomic analysis and model construction. (3) Machine learning models, including Bagging DecisionTree, XGBOOST, Random Forest, Support Vector Machine (SVM), and Quadratic Discriminant Analysis (QDA), are constructed Model 1 (RS model). The patients were divided into training set (*n* = 163) and test set (*n* = 40) according to the ratio of 8:2.

### 2.6 Statistical analysis

The data analysis was performed using SPSS 25.0, MedCalc 22.014, and uAI Research Portal software (version 1.1, United, China). Kolmogorov–Smirnov was used to test the normality of measurement data. Continuous variables were expressed as mean ± SDs or median and interquartile range as appropriate. Categorical variables were reported as count and percentage. Continuous variables were compared with the Student *t*-test or Mann–Whitney test. Categorical variables were compared using χ2 or the Fisher exact test. Univariate logistic regression was employed to analyze the correlation between the traditional features of each patient and symptomatic plaques. Features with *p* < 0.05 in the univariate logistic regression were included in the multivariate logistic regression analysis for further analysis.

**Figure 3:**
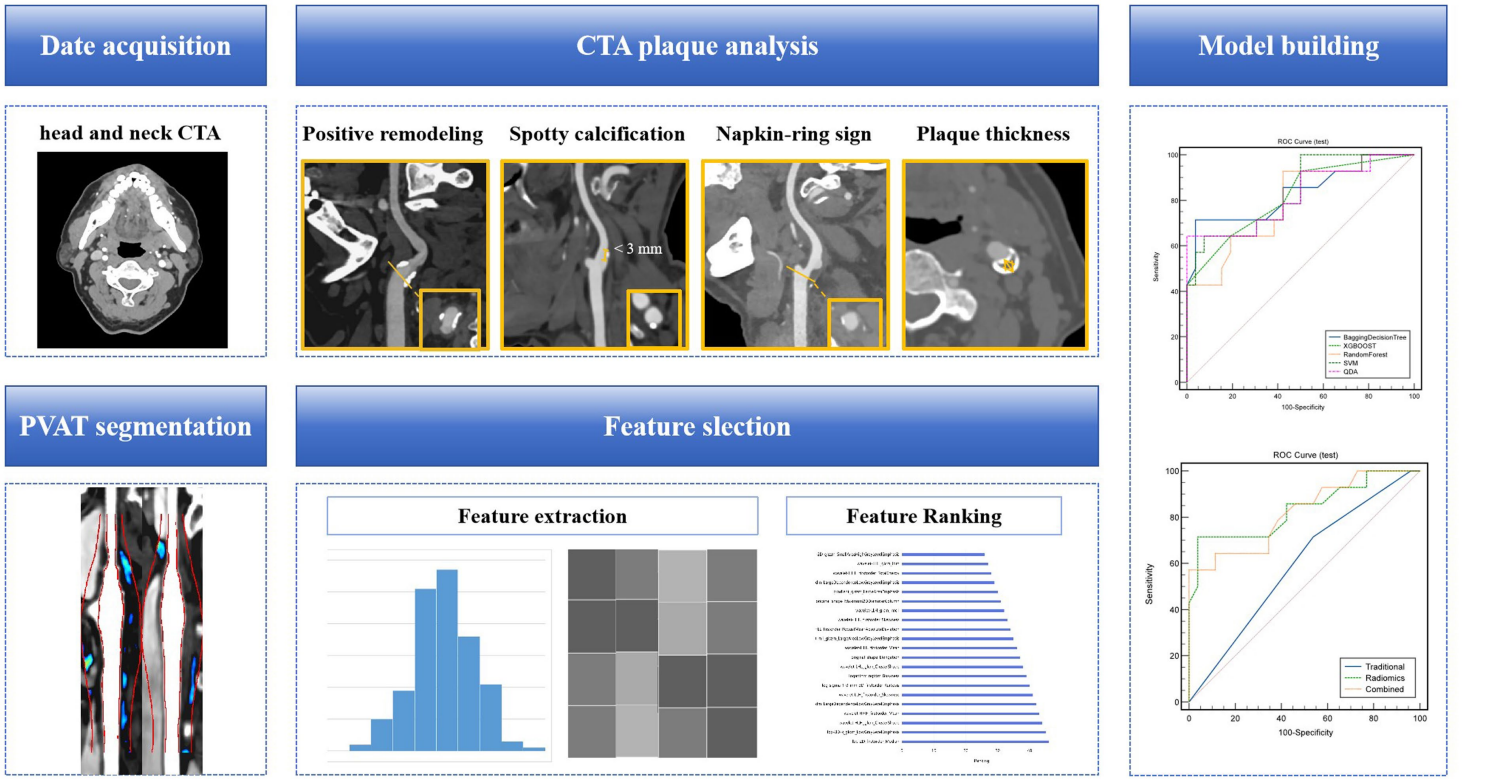
A flow chart of the model development process. Collect clinical and radiological information of patients, analyze and extract traditional features of carotid artery plaques. Based on head and neck CTA, segment the PVAT of carotid artery plaques, extract radiomics features, and use algorithms such as minimal redundancy maximal relevance, recursive feature elimination, and linear discriminant analysis for feature selection and dimensionality reduction. Construct a machine learning model for identifying symptomatic plaques using radiomics features, traditional features (clinical + traditional radiological features), and a combination of radiomics and traditional features. CTA, computed tomography angiography; PVAT, perivascular adipose tissue.

In order to investigate if carotid PVAT imaging RS provides additional value in diagnosing symptomatic plaques compared to traditional plaque analysis, two models were developed. Model 2 (Traditional model) included different clinical and conventional CTA imaging features between symptomatic and asymptomatic patient groups in a multivariate logistic regression analysis. Model 3 (Combined model): Model 2 was enhanced by incorporating the Model 1. The machine learning algorithm parameters used in both models were identical to those in Model 1. Area Under the Curve (AUC) was used to evaluate the ability of the two groups of models to identify symptomatic plaques. The deLong test was used to compare the differences between AUCs. *p* < 0.05 was considered statistically significant (Figure 3).

## 3 Results

### 3.1 Characteristics of the study patients

This study included a total of 203 patients, with an average age of 71.87 ± 9.63 years and a total of 115 men. Among them, there were 71 cases in the symptomatic group and 132 cases in the asymptomatic group.

In the multivariate logistic regression analysis, it was found that the proportion of positive remodeling in the symptomatic group was higher than that in the asymptomatic group (97.2% vs. 84.8%, *p* = 0.017). Additionally, the proportion of statin use in the symptomatic group was significantly lower than that in the asymptomatic group (15.5% vs. 47%, *p* < 0.001).

Other factors such as age, gender, BMI, history of hypertension, diabetes mellitus, hyperlipidemia, smoking history, history of antihypertensive drugs, history of antiplatelet drugs, plaque location, degree of plaque stenosis, plaque length, plaque thickness, remodeling index, FAI at the most stenosis of the plaque, FAI within the stenosis of the plaque, conformal remodeling, low-density plaque, punctate calcification, napkin ring sign, and high-risk plaque distribution, plaque type, and plaque ulcer did not show statistically significant differences in the multivariate regression analysis (*p* > 0.05) (Table 1).

**Table 1:** Traditional characteristics of the patient.

Characteristic	ALL patients	Symptomatic	Asymptomatic	Univariate analysis	Multivariate	
					OR (95% CI)	*p* value
Clinical characteristics						
Age, y, mean ± SD	71.87 ± 9.63	72.38 ± 9.54	71.59 ± 9.7	0.579		
No. of men, *n* (%)	115 (56.7)	39 (54.9)	76 (57.6)	0.717		
BMI, kg/m2, mean ± SD	23.34 ± 3.12	22.8 ± 2.83	23.63 ± 3.24	0.073		
Risk factors						
Hypertension, *n* (%)	155 (76.4)	55 (77.5)	100 (75.8)	0.785		
Diabetes mellitus, *n* (%)	72 (35.5)	28 (39.4)	44 (33.3)	0.386		
Hyperlipidemia, *n* (%)	50 (24.6)	19 (26.8)	31 (23.5)	0.605		
Smoking, *n* (%)	55 (27.1)	24 (33.8)	31 (23.5)	0.115		
History of medications						
Antihypertension use, *n* (%)	155 (76.4)	55 (77.5)	100 (75.8)	0.785		
Statin use, *n* (%)	73 (36)	11 (15.5)	62 (47)	< 0.001	4.950 (2.336, 10.492)	<0.001
Antiplatelet use, *n* (%)	90 (44.3)	32 (45.1)	58 (43.9)	0.877		
Quantitative plaque characteristics						
Diameter stenosis, %, mean ± SD	34.31 ± 22.95	32.08 ± 21.55	35.51 ± 23.67	0.31		
Lesion length, mm, mean ± SD	1.08 ± 7.68	1.02 ± 7.53	11.07 ± 7.78	0.462		
Plaque thickness, mm, mean ± SD	3.45 ± 1.47	3.25 ± 1.28	3.56 ± 1.56	0.154		
Remodeling index, mean ± SD	1.40 ± 0.25	1.43 ± 0.25	1.39 ± 0.25	0.224		
Stenosis FAI, HU, mean ± SD	−65.29 ± 13.83	−63.02 ± 14.31	−66.51 ± 13.46	0.087		
Stenosis range FAI, HU, mean ± SD	−66.06 ± 0.89	−63.99 ± 12.87	−67.18 ± 12.41	0.086		
Quantitative plaque characteristics						
Positive remodeling, *n* (%)	181 (89.2)	69 (97.2)	112 (84.8)	0.016	0.102 (0.016, 0.671)	0.017
Low-attenuation plaque, *n* (%)	23 (11.3)	10 (14.1)	13 (9.8)	0.366		
Spotty calcification, *n* (%)	129 (63.5)	54 (76.1)	75 (56.8)	0.007	0.220 (0.040, 1.205)	0.081
Napkin-ring sign, *n* (%)	10 (4.9)	3 (4.2)	7 (5.3)	0.736		
High-risk plaque, *n* (%)	126 (62.1)	54 (76.1)	72 (54.5)	0.003	1.839 (0.328, 10.308)	0.488
Plaque ulcer, *n* (%)	39 (19.2)	13 (18.3)	26 (19.7)	0.811		
Plaque location(L), *n* (%)	100 (49.3)	40 (56.3)	60 (45.5)	0.139		
Plaque Type, *n* (%)				0.773		
Calcified plaque, *n* (%)	114 (56.2)	42 (59.2)	72 (54.5)			
Non-calcified plaque, *n* (%)	14 (6.9)	4 (5.6)	10 (7.6)			
Mixed plaque, *n* (%)	75 (36.9)	25 (35.2)	50 (37.9)			

BMI, body mass index. Data are displayed as mean (SD) or number (percent).

### 3.2 RS model of carotid PVAT

**Figure 4:**
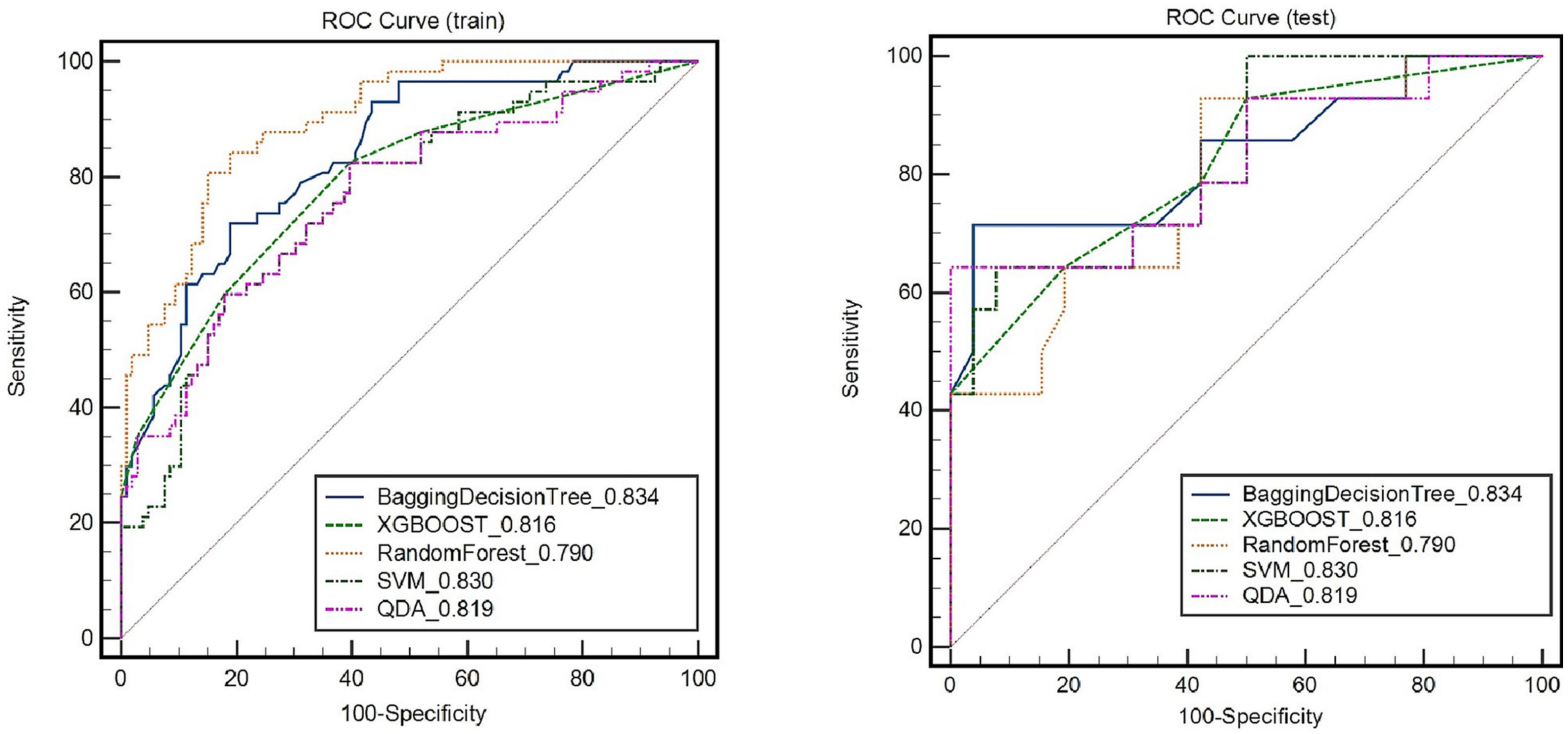
Receiver operating characteristic (ROC) curves of radiomic signature in identifying symptomatic plaques in different models.

The RS model showed the highest diagnostic performance in identifying symptomatic plaques within the Bagging Decision Tree model, achieving an AUC of 0.837 (95%CI: 0.775, 0.899) in the training set and an AUC of 0.834 (95%CI: 0.685, 0.982) in the testing set. These results were significantly better than the performances of the XGBOOST, Random Forest, SVM, and QDA models (*p* < 0.05) (Figure 4; Table 2).

**Table 2:** Predictive ability of all radiomics models.

Model	Training cohort				Testing cohort			
	AUC (95% CI)	SPE	SEN	ACC	AUC (95% CI)	SPE	SEN	ACC
Bagging decision-tree	0.837 (0.775, 0.899)	0.811	0.702	0.773	0.834 (0.685, 0.982)	0.731	0.714	0.725
XGBOOST	0.79 (0.717, 0.863)	0.821	0.596	0.742	0.816 (0.675, 0.957)	0.808	0.643	0.75
Random forest	0.897 (0.85, 0.944)	0.849	0.754	0.816	0.79 (0.64, 0.94)	0.808	0.643	0.75
SVM	0.762 (0.685, 0.839)	0.774	0.614	0.718	0.83 (0.694, 0.965)	0.643	0.769	0.725
QDA	0.765 (0.686, 0.843)	0.821	0.698	0.687	0.819 (0.665, 0.972)	0.714	0.692	0.7

AUC, area under the curve.

### 3.3 RS combined with the traditional model

**Figure 5:**
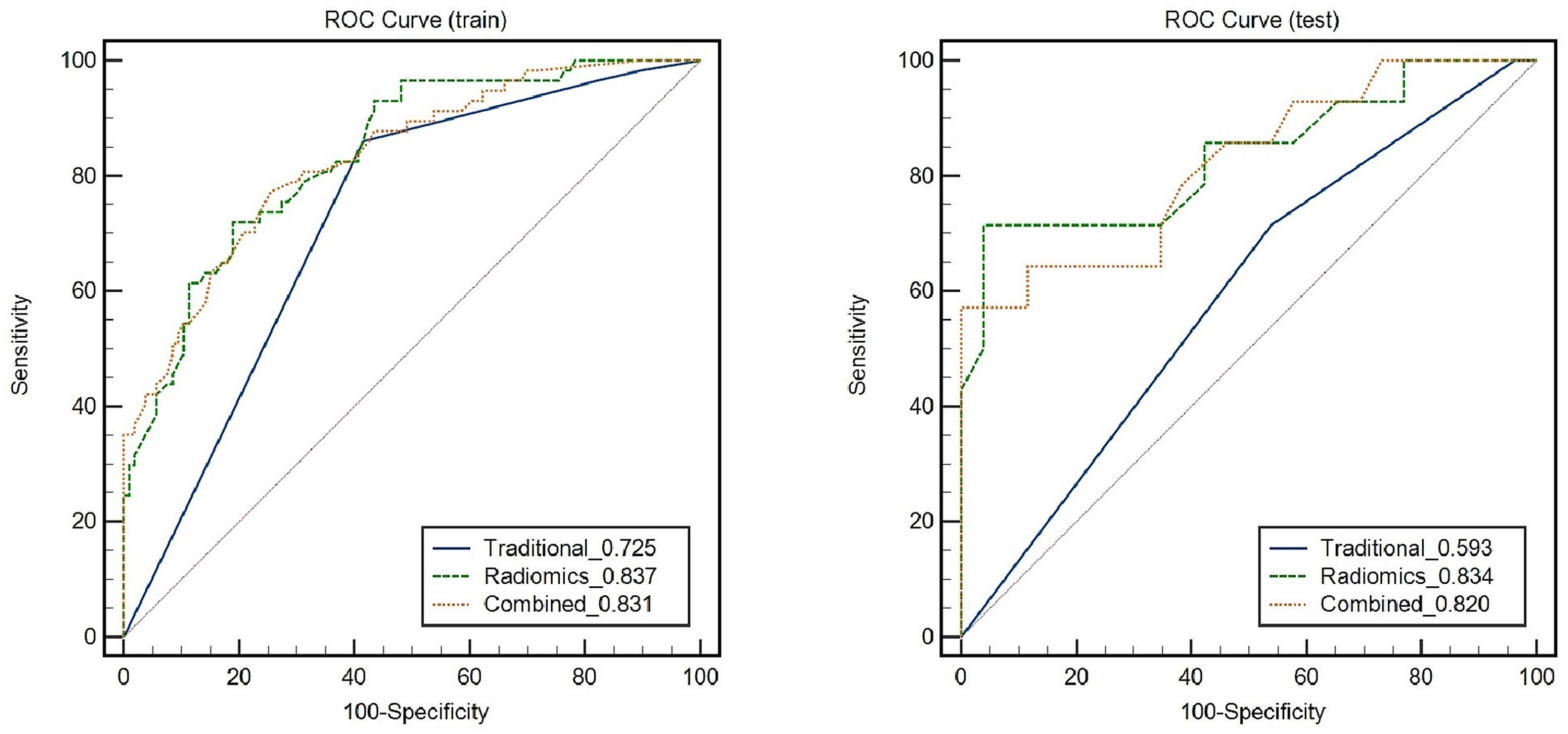
Receiver Operating Characteristic (ROC) curves demonstrate the diagnostic performance of symptomatic plaques in different feature sets.

Figure 5 depicts the diagnostic performance of traditional feature models and the RS model in identifying symptomatic plaques across different sets of data. In the training set, the traditional feature model achieved an AUC of 0.725 (95%CI: 0.695, 0.791), while in the testing set, the AUC was 0.593 (95%CI: 0.438, 0.749). Upon incorporating the RS model into the traditional feature model, the AUC in the training set improved to 0.831 (95%CI: 0.765, 0.896), and in the testing set, it reached 0.82 (95%CI: 0.675, 0.965).

Through the Delong test, it was determined that the combination of the RS model with the traditional feature model yielded a significantly higher AUC for distinguishing symptomatic plaques compared to using the traditional model alone (AUC: 0.82 vs. AUC: 0.593; Z = 2.822, *p* = 0.0048). Furthermore, when used independently, the RS model demonstrated a superior AUC in differentiating symptomatic plaques compared to the traditional model (AUC: 0.834 vs. AUC: 0.593; Z = 2.114, *p* = 0.0345).

## 4 Discussion

This study confirms that the RS model, based on carotid PVAT, has demonstrated significant improvement over the current traditional models in distinguishing symptomatic plaques. The RS model, relying on carotid PVAT, exhibited a higher AUC in the discrimination of symptomatic plaques (AUC: 0.834; 95% CI: 0.685, 0.982), compared to the traditional model (AUC: 0.593; 95% CI: 0.438, 0.749).

CT-based radiomics has been shown to be able to accurately classify diseases by extracting a large number of quantitative radiomics features that are invisible to the human eye (27). In this study, the diagnostic performance of the RS model, based on carotid PVAT, in distinguishing symptomatic plaques was evaluated in both the training set (AUC: 0.837; 95% CI: 0.775, 0.899) and the testing set (AUC: 0.834; 95% CI: 0.685, 0.982). In a study conducted by Chen et al. (4), which included 60 symptomatic and 84 asymptomatic individuals based on the occurrence of ischemic stroke or TIA within a 2 week period, the RS model based on carotid plaque PVAT demonstrated an AUC of 0.740 (95% CI: 0.644, 0.835) in the training set and 0.618 (95% CI: 0.440, 0.794) in the testing set (4). The relatively higher ROC performance observed in our study compared to Chen et al. may be attributed to the fact that Chen et al. extracted PVAT pixel values from the surrounding adipose tissue around the maximum stenosis level of the carotid plaque, resulting in fewer PVAT pixel values and a 2D image. Consequently, the performance of their RS model was relatively lower.

Recently, progress has been made in utilizing carotid plaque RS to differentiate symptomatic plaques. Xia et al. conducted a study on 179 patients with 219 carotid plaques, stratifying them into a TIA group and a non-TIA group according to the presence or absence of TIA after CTA examination (28). Their RS model for distinguishing the TIA group yielded a maximum AUC of 0.746 in the testing set. Our study shows an RS model based on carotid PVAT with a testing set AUC of 0.834, thus indicating superior diagnostic performance compared to Xia et al.’s study on a carotid plaque RS model. This suggests that the carotid PVAT-based RS model has the potential to provide additional benefits in identifying symptomatic plaques.

A large study showed (29) that long-term achievement of low LDL-C levels, as low as less than 20 mg per deciliter (<0.5 mmol per liter), was associated with a reduced risk of cardiovascular outcomes without significant safety concerns in patients with atherosclerotic cardiovascular and cerebrovascular disease. The lower proportion of statin use in the symptomatic group than in the asymptomatic group in this study may be due to the higher incidence of ischemic stroke or TIA in patients who do not receive statin therapy.

There was no significant difference in FAI at the narrowest point of the plaque and within the stenosis of the plaque between the two groups, which may be related to the fact that most of the patients in this study were elderly with an average age of 71.87 ± 9.63 years. FAI is used to dynamically monitor vascular inflammation (5) by measuring the mean density of adipose tissue on CT to reflect the change in lipid content. The patients in this study have a long history of atherosclerosis, PVAT of carotid plaque has gone into the chronic phase, lipid fibrosis and microvascular remodeling occur, and the dynamic change of lipid content is small (11, 30), so the ability of FAI to dynamically monitor vascular inflammation is limited (11). At the same time, Serum C reactive protein (CRP) is a marker of systemic inflammation and is associated with an increased risk of stroke and unstable carotid atherosclerotic plaques (31). However, high-sensitivity CRP is usually driven by other inflammatory conditions such as infection, arthritis, etc., and cannot specifically reflect the local inflammation of carotid atherosclerosis. PET is considered to be the most reliable non-invasive imaging modality for vascular inflammation. However, its clinical application is limited due to its low spatial resolution, high radiation exposure, and high cost. In our study, the RS of carotid PVAT was available, and the diagnostic efficacy of the RS model of carotid PVAT in identifying symptomatic plaques was 0.745. RS analysis can accurately capture the texture changes of PVAT and reflect the level of vascular inflammation.

The carotid plaque PVAT extracted in this study was extended 2 cm above and below the center of the carotid segment bifurcation, with a total of 4 cm as the longitudinal measurement distance. Because of the vascular shear stress (32), the vast majority of extracranial carotid plaques were distributed at the carotid bifurcation, and the plaques of the cases included in this study were distributed within 2 cm above and below the carotid bifurcation. Second, there was little fat distribution around the carotid artery, and the plaques of the cases included in this study were distributed in the range of 2 cm above and below the carotid bifurcation. This study referred to the method of extracting the proximal 4 cm PVAT of the coronary artery with peri-coronary fat and appropriately increased the collection range of the PVAT of carotid plaques to ensure the accuracy of RS extraction in PVAT. Third, Oikonomou et al. (25) showed that perivascular FAI at 4 cm proximal to the right coronary artery can reflect global coronary inflammation and predict cardiac mortality. In our study, PVAT at 4 cm of the carotid bifurcation also has the potential to represent the risk of vascular inflammation at the carotid bifurcation plaque and the whole carotid artery segment.

This paper has the following limitations: (1) This paper adopts the mainstream method used in current related research to identify symptomatic plaques, but it lacks a gold standard. In the future, we aim to collect plaque samples through carotid artery stripping and other procedures to accurately identify culprit plaques; (2) The lack of external validation datasets to evaluate the diagnostic efficacy of machine learning models; and (3) As symptomatic and asymptomatic determination of plaques happens before CTA exams, it would have selection bias towards the model performance in the real clinical settings. In the next step of our research, we will conduct a prospective study on patients undergoing head and neck CTA to explore the association between PVAT imaging-based radiomics of carotid plaques and the occurrence of acute ischemic cerebrovascular events.

## 5 Conclusion

The RS model of carotid plaque PVAT, when combined with the traditional feature model, demonstrates a significant improvement in the diagnostic performance for identifying symptomatic plaques compared to the traditional feature model alone. This indicates that the RS model of carotid plaque PVAT can serve as an objective indicator for evaluating plaque risk, providing a basis for risk stratification, as well as the diagnosis and treatment of carotid atherosclerotic diseases.

## Data availability statement

The raw data supporting the conclusions of this article will be made available by the authors, without undue reservation.

## Ethics statement

The studies involving humans were approved by Shunde Hospital of Guangzhou University of Chinese Medicine. The studies were conducted in accordance with the local legislation and institutional requirements. The participants provided their written informed consent to participate in this study. Written informed consent was obtained from the individual(s) for the publication of any potentially identifiable images or data included in this article.

## Author contributions

J-YN: Conceptualization, Data curation, Formal analysis, Investigation, Methodology, Writing – original draft. W-XC: Data curation, Formal analysis, Methodology, Writing – review & editing. ZZ: Writing – review & editing. M-YZ: Data curation, Formal analysis, Methodology, Writing – review & editing. Y-JZ: Data curation, Funding acquisition, Supervision, Writing – review & editing. Q-DW: Conceptualization, Funding acquisition, Methodology, Writing – review & editing.
