# Corrigendum: Initial experience with radiomics of carotid perivascular adipose tissue in identifying symptomatic plaque

**DOI:** 10.3389/fneur.2024.1509324

**Published:** 2024-10-29

**Authors:** Ji-Yan Nie, Wen-Xi Chen, Zhi Zhu, Ming-Yu Zhang, Yu-Jin Zheng, Qing-De Wu

**Affiliations:** ^1^Department of Radiology, ShunDe Hospital of Guangzhou University of Chinese Medicine, Foshan, China; ^2^Graduate School, Guangzhou University of Chinese Medicine, Guangzhou, China

**Keywords:** carotid atherosclerosis, perivascular adipose tissue, radiomics, stroke, transient ischemic attack

In the published article, there was an error in affiliation [1]. Instead of “^1^Department of Radiology, Shunde Hospital of Guangzhou University of Traditional Chinese Medicine, Shunde, China,” it should be “^1^Department of Radiology, ShunDe Hospital of Guangzhou University of Chinese Medicine, Foshan, China.”

The authors apologize for this error and state that this does not change the scientific conclusions of the article in any way. The original article has been updated.

